# 2-Amino-5-chloro­pyridinium 6-oxo-1,6-dihydro­pyridine-2-carboxyl­ate 0.85-hydrate

**DOI:** 10.1107/S1600536810032307

**Published:** 2010-08-18

**Authors:** Madhukar Hemamalini, Hoong-Kun Fun

**Affiliations:** a X-ray Crystallography Unit, School of Physics, Universiti Sains Malaysia, 11800 USM, Penang, Malaysia

## Abstract

In the title salt, C_5_H_6_ClN_2_^+^·C_6_H_4_NO_3_^−^·0.85H_2_O, the pyridin­ium ring is planar, with a maximum deviation of 0.010 (2) Å. In the crystal structure, the cations, anions and water mol­ecules are linked *via* N—H⋯O, O—H⋯O and C—H⋯O hydrogen bonds, forming a three-dimensional network.

## Related literature

For applications of inter­molecular inter­actions, see: Braga *et al.* (2002 ▶); Lam & Mak (2000 ▶). For related structures, see: Hemamalini & Fun (2010*a* ▶,*b* ▶,*c* ▶,*d* ▶,*e* ▶,*f* ▶); Sawada & Ohashi (1998 ▶). For hydrogen-bond motifs, see: Bernstein *et al.* (1995 ▶).
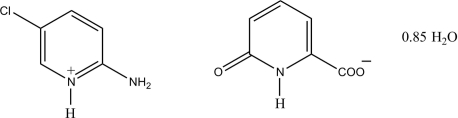


## Experimental

### 

#### Crystal data

C_5_H_6_ClN_2_^+^·C_6_H_4_NO_3_^−^·0.85H_2_O
*M* *_r_* = 282.98
Orthorhombic, 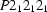

*a* = 3.8096 (1) Å
*b* = 15.6046 (3) Å
*c* = 20.9370 (3) Å
*V* = 1244.65 (4) Å^3^
*Z* = 4
Mo *K*α radiation
μ = 0.32 mm^−1^
*T* = 296 K
0.52 × 0.22 × 0.11 mm

#### Data collection

Bruker SMART APEXII CCD area-detector diffractometer
Absorption correction: multi-scan (*SADABS*; Bruker, 2009 ▶) *T*_min_ = 0.851, *T*_max_ = 0.966
15196 measured reflections
3632 independent reflections
3129 reflections with *I* > 2σ(*I*)
*R*_int_ = 0.026

#### Refinement

*R*[*F* ^2^ > 2σ(*F* ^2^)] = 0.036
*wR*(*F* ^2^) = 0.113
*S* = 1.10
3632 reflections
215 parameters
H atoms treated by a mixture of independent and constrained refinement
Δρ_max_ = 0.20 e Å^−3^
Δρ_min_ = −0.17 e Å^−3^
Absolute structure: Flack (1983 ▶), with 1458 Fridel pairs
Flack parameter: −0.04 (6)

### 

Data collection: *APEX2* (Bruker, 2009 ▶); cell refinement: *SAINT* (Bruker, 2009 ▶); data reduction: *SAINT*; program(s) used to solve structure: *SHELXTL* (Sheldrick, 2008 ▶); program(s) used to refine structure: *SHELXTL*; molecular graphics: *SHELXTL*; software used to prepare material for publication: *SHELXTL* and *PLATON* (Spek, 2009 ▶).

## Supplementary Material

Crystal structure: contains datablocks global, I. DOI: 10.1107/S1600536810032307/ci5153sup1.cif

Structure factors: contains datablocks I. DOI: 10.1107/S1600536810032307/ci5153Isup2.hkl

Additional supplementary materials:  crystallographic information; 3D view; checkCIF report

## Figures and Tables

**Table 1:** Hydrogen-bond geometry (Å, °)

*D*—H⋯*A*	*D*—H	H⋯*A*	*D*⋯*A*	*D*—H⋯*A*
O1*W*—H1*W*1⋯O3^i^	0.85	1.85	2.696 (3)	174
O1*W*—H2*W*1⋯O1*W*^ii^	0.85	1.99	2.732 (5)	145
N1—H1N1⋯O3^iii^	0.98 (2)	1.67 (2)	2.637 (2)	170 (2)
N2—H1N2⋯O1^iv^	0.87 (2)	1.97 (2)	2.823 (2)	168 (2)
N2—H2N2⋯O2^iii^	0.84 (3)	2.04 (3)	2.882 (2)	179 (3)
C4—H4⋯O1^v^	0.93 (2)	2.39 (2)	3.296 (2)	166 (2)

Symmetry codes: (i) 
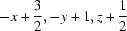
; (ii) 
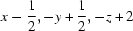
; (iii) 
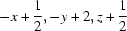
; (iv) 
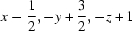
; (v) 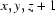.

**Fig. 1.**
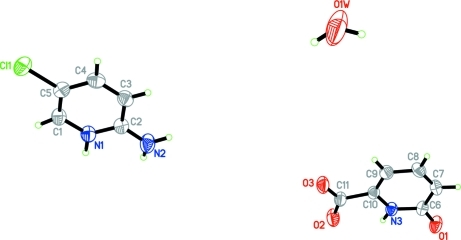
The asymmetric unit of the title compound. Displacement ellipsoids are drawn at the 50% probability level.

**Fig. 2.**
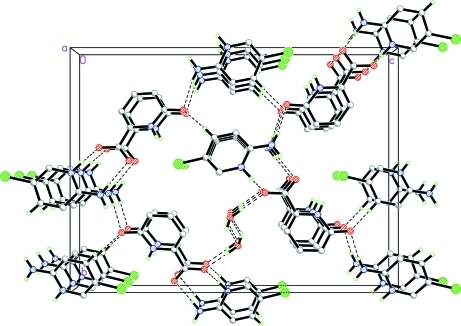
The crystal packing of the title compound, showing part of a hydrogen-bonded network. H atoms not involved in hydrogen bonding (dashed lines) have been omitted for clarity.

## supplementary crystallographic information

### Comment

Intermolecular interaction analyses in crystalline systems are very important in supramolecular chemistry (Braga *et al.*, 2002). These interactions are responsible for crystal packing, and through an understanding of such interactions we can comprehend collective properties and design new crystals with specific physical and chemical properties (Lam & Mak, 2000). We have been interested in hydrogen-bonded systems formed by 2-amino pyridines and carboxylic acids that generate molecular assemblies (Hemamalini & Fun, 2010*a*,*b*,*c*,*d*,*e*,*f*). In continuation of our studies of pyridinium derivatives, the crystal structure determination of the title compound has been undertaken.

The asymmetric unit, (Fig. 1), contains one 2-amino-5-chloropyridinium cation, one 6-oxo-1,6-dihydropyridine-2-carboxylate anion and one water molecule with a refined site occupany of 0.85. The pyridinium ring is essentially planar, with a maximum deviation of 0.010 (2) Å for atom C5. In the 2-amino-5-chloropyridinium cation, a wider than normal angle [C1—N1—C2 = 122.55 (14)°] is subtended at the protonated N1 atom. The anion exists in the keto–enol tautomerism of the -CONH moiety. Similar tautomerism is also observed in the crystal structure of 2-oxo-1,2-dihydropyridine-6-carboxylic acid (Sawada & Ohashi, 1998).

In the crystal packing (Fig. 2), the protonated N1 atom and the 2-amino group (N2) are hydrogen-bonded to the carboxylate oxygen atoms (O2 and O3) via a pair of intermolecular N1—H1N1···O3 and N2—H2N2···O2 hydrogen bonds, forming an *R*_2_^2^(8) ring motif (Bernstein *et al.*, 1995). The ion pairs are further connected via O1W—H1W1···O3, O1W—H2W1···O1W, N2—H1N2···O1 and C4—H4···O1 (Table 1) hydrogen bonds, forming a three-dimensional network. The crystal of title compound is isomorphous with that of 2-amino-5-bromopyridinium 6-oxo-1,6-dihydropyridine-2-carboxylate monohydrate (Hemamalini & Fun, 2010*f*).

### Experimental

A hot methanol solution (20 ml) of 2-amino-5-chloropyridine (64 mg, Aldrich) and 6-hydroxypicolinic acid (69 mg, Merck) were mixed and warmed over a heating magnetic stirrer hotplate for a few minutes. The resulting solution was allowed to cool slowly at room temperature and crystals of the title compound appeared after a few days.

### Refinement

The site occupancy of the water molecule was initially refined and then fixed at 0.85 in the final refinement. The H-atoms were located in a difference Fourier map and refined freely [ranges of C—H = 0.89 (2)–0.95 (2) Å and N—H = 0.82 (3)–0.97 (2) Å]. The water H atoms were allowed to ride on the parent O atom.

### Crystal data

**Table:**

C_5_H_6_ClN_2_^+^·C_6_H_4_NO_3_^−^·0.85H_2_O	*F*(000) = 586
*M**_r_* = 282.98	*D*_x_ = 1.510 Mg m^−^^3^
Orthorhombic, *P*2_1_2_1_2_1_	Mo *K*α radiation, λ = 0.71073 Å
Hall symbol: P 2ac 2ab	Cell parameters from 6806 reflections
*a* = 3.8096 (1) Å	θ = 2.3–29.0°
*b* = 15.6046 (3) Å	µ = 0.32 mm^−^^1^
*c* = 20.9370 (3) Å	*T* = 296 K
*V* = 1244.65 (4) Å^3^	Needle, green
*Z* = 4	0.52 × 0.22 × 0.11 mm

### Data collection

**Table:**

Bruker SMART APEXII CCD area-detector diffractometer	3632 independent reflections
Radiation source: fine-focus sealed tube	3129 reflections with *I* > 2σ(*I*)
graphite	*R*_int_ = 0.026
φ and ω scans	θ_max_ = 30.1°, θ_min_ = 1.6°
Absorption correction: multi-scan (*SADABS*; Bruker, 2009)	*h* = −4→5
*T*_min_ = 0.851, *T*_max_ = 0.966	*k* = −21→21
15196 measured reflections	*l* = −29→29

### Refinement

**Table:**

Refinement on *F*^2^	Secondary atom site location: difference Fourier map
Least-squares matrix: full	Hydrogen site location: inferred from neighbouring sites
*R*[*F*^2^ > 2σ(*F*^2^)] = 0.036	H atoms treated by a mixture of independent and constrained refinement
*wR*(*F*^2^) = 0.113	*w* = 1/[σ^2^(*F*_o_^2^) + (0.0653*P*)^2^ + 0.0443*P*] where *P* = (*F*_o_^2^ + 2*F*_c_^2^)/3
*S* = 1.10	(Δ/σ)_max_ = 0.001
3632 reflections	Δρ_max_ = 0.20 e Å^−^^3^
215 parameters	Δρ_min_ = −0.17 e Å^−^^3^
0 restraints	Absolute structure: Flack (1983), with 1458 Fridel pairs
Primary atom site location: structure-invariant direct methods	Flack parameter: −0.04 (6)

### Special details

**Table:**

Geometry. All s.u.'s (except the s.u. in the dihedral angle between two l.s. planes) are estimated using the full covariance matrix. The cell s.u.'s are taken into account individually in the estimation of s.u.'s in distances, angles and torsion angles; correlations between s.u.'s in cell parameters are only used when they are defined by crystal symmetry. An approximate (isotropic) treatment of cell s.u.'s is used for estimating s.u.'s involving l.s. planes.
Refinement. Refinement of F^2^ against ALL reflections. The weighted R-factor wR and goodness of fit S are based on F^2^, conventional R-factors R are based on F, with F set to zero for negative F^2^. The threshold expression of F^2^ > 2σ(F^2^) is used only for calculating R-factors(gt) etc. and is not relevant to the choice of reflections for refinement. R-factors based on F^2^ are statistically about twice as large as those based on F, and R- factors based on ALL data will be even larger.

### Fractional atomic coordinates and isotropic or equivalent isotropic displacement parameters (Å^2^)

**Table:**

	*x*	*y*	*z*	*U*_iso_*/*U*_eq_	Occ. (<1)
Cl1	0.29968 (15)	0.97559 (3)	1.16268 (2)	0.04826 (15)	
N1	0.0504 (4)	0.98883 (9)	0.97983 (7)	0.0358 (3)	
N2	0.1288 (6)	0.90253 (11)	0.89193 (7)	0.0473 (4)	
C1	0.0873 (5)	1.00748 (10)	1.04320 (8)	0.0365 (4)	
C2	0.1708 (5)	0.91546 (9)	0.95385 (8)	0.0339 (3)	
C3	0.3321 (6)	0.85531 (10)	0.99503 (8)	0.0378 (4)	
C4	0.3690 (5)	0.87223 (11)	1.05852 (9)	0.0389 (4)	
C5	0.2467 (5)	0.95094 (10)	1.08254 (7)	0.0361 (4)	
O1	0.8680 (5)	0.75705 (8)	0.15429 (5)	0.0483 (4)	
O2	0.6524 (5)	0.96179 (8)	0.31378 (6)	0.0527 (4)	
O3	0.8337 (5)	0.90935 (8)	0.40756 (6)	0.0516 (4)	
N3	0.8628 (4)	0.82238 (9)	0.25126 (6)	0.0332 (3)	
C6	0.9424 (5)	0.75403 (10)	0.21242 (8)	0.0363 (4)	
C7	1.1066 (5)	0.68363 (11)	0.24435 (9)	0.0397 (4)	
C8	1.1756 (6)	0.68705 (11)	0.30786 (9)	0.0414 (4)	
C9	1.0842 (6)	0.75963 (12)	0.34448 (8)	0.0398 (4)	
C10	0.9247 (5)	0.82601 (10)	0.31526 (8)	0.0321 (3)	
C11	0.7932 (6)	0.90675 (10)	0.34770 (7)	0.0386 (4)	
O1W	0.5775 (11)	0.18936 (16)	1.01198 (10)	0.1069 (12)	0.85
H1W1	0.5888	0.1576	0.9790	0.101 (14)*	0.85
H2W1	0.4401	0.2303	1.0216	0.109 (17)*	0.85
H1	−0.012 (6)	1.0566 (13)	1.0558 (9)	0.040 (5)*	
H3	0.416 (6)	0.8069 (14)	0.9779 (9)	0.044 (6)*	
H4	0.474 (6)	0.8348 (12)	1.0870 (8)	0.032 (5)*	
H8	1.274 (6)	0.6427 (13)	0.3299 (9)	0.049 (6)*	
H9	1.141 (9)	0.7623 (15)	0.3865 (11)	0.065 (7)*	
H7	1.155 (7)	0.6344 (12)	0.2193 (9)	0.042 (5)*	
H1N1	−0.068 (6)	1.0297 (13)	0.9520 (10)	0.046 (6)*	
H1N2	0.222 (7)	0.8578 (14)	0.8740 (10)	0.048 (6)*	
H2N2	0.050 (7)	0.9426 (17)	0.8691 (11)	0.057 (7)*	
H1N3	0.771 (6)	0.8649 (13)	0.2351 (9)	0.043 (6)*	

### Atomic displacement parameters (Å^2^)

**Table:**

	*U*^11^	*U*^22^	*U*^33^	*U*^12^	*U*^13^	*U*^23^
Cl1	0.0578 (3)	0.0516 (2)	0.0353 (2)	−0.0024 (2)	−0.0049 (2)	−0.00042 (17)
N1	0.0431 (8)	0.0311 (6)	0.0331 (7)	0.0030 (6)	0.0011 (6)	0.0030 (5)
N2	0.0696 (13)	0.0352 (7)	0.0370 (7)	0.0118 (8)	−0.0039 (8)	−0.0020 (6)
C1	0.0429 (9)	0.0308 (7)	0.0358 (8)	0.0003 (7)	0.0029 (7)	0.0006 (6)
C2	0.0368 (8)	0.0298 (7)	0.0352 (7)	−0.0011 (7)	0.0018 (8)	0.0024 (6)
C3	0.0400 (9)	0.0302 (7)	0.0432 (8)	0.0033 (7)	−0.0002 (8)	0.0020 (6)
C4	0.0372 (9)	0.0358 (8)	0.0435 (9)	0.0001 (8)	−0.0030 (8)	0.0099 (7)
C5	0.0370 (9)	0.0383 (8)	0.0329 (7)	−0.0048 (7)	−0.0002 (7)	0.0029 (6)
O1	0.0731 (10)	0.0412 (6)	0.0306 (5)	0.0021 (7)	−0.0036 (7)	−0.0059 (5)
O2	0.0771 (11)	0.0396 (6)	0.0414 (6)	0.0175 (7)	−0.0040 (8)	−0.0052 (5)
O3	0.0709 (10)	0.0500 (7)	0.0339 (6)	0.0190 (8)	−0.0034 (8)	−0.0099 (5)
N3	0.0434 (8)	0.0265 (6)	0.0297 (6)	0.0026 (6)	−0.0020 (6)	0.0002 (5)
C6	0.0427 (9)	0.0324 (7)	0.0338 (7)	−0.0044 (7)	0.0038 (8)	−0.0036 (6)
C7	0.0440 (10)	0.0321 (7)	0.0429 (9)	0.0048 (8)	0.0060 (8)	−0.0045 (7)
C8	0.0445 (10)	0.0368 (8)	0.0430 (9)	0.0099 (8)	0.0017 (9)	0.0057 (7)
C9	0.0447 (10)	0.0421 (8)	0.0325 (8)	0.0040 (8)	−0.0020 (8)	0.0005 (7)
C10	0.0330 (8)	0.0334 (7)	0.0300 (7)	−0.0012 (7)	0.0024 (7)	−0.0026 (6)
C11	0.0466 (10)	0.0357 (7)	0.0335 (7)	0.0030 (8)	−0.0001 (8)	−0.0054 (6)
O1W	0.187 (4)	0.0767 (15)	0.0574 (12)	0.013 (2)	−0.0226 (18)	−0.0259 (11)

### Geometric parameters (Å, °)

**Table:**

Cl1—C5	1.7331 (16)	O2—C11	1.237 (2)
N1—C2	1.348 (2)	O3—C11	1.2633 (18)
N1—C1	1.365 (2)	N3—C10	1.362 (2)
N1—H1N1	0.97 (2)	N3—C6	1.375 (2)
N2—C2	1.322 (2)	N3—H1N3	0.82 (2)
N2—H1N2	0.87 (2)	C6—C7	1.430 (3)
N2—H2N2	0.84 (3)	C7—C8	1.356 (3)
C1—C5	1.351 (2)	C7—H7	0.95 (2)
C1—H1	0.90 (2)	C8—C9	1.411 (3)
C2—C3	1.415 (2)	C8—H8	0.91 (2)
C3—C4	1.363 (3)	C9—C10	1.348 (2)
C3—H3	0.89 (2)	C9—H9	0.91 (2)
C4—C5	1.407 (2)	C10—C11	1.516 (2)
C4—H4	0.925 (19)	O1W—H1W1	0.85
O1—C6	1.2506 (19)	O1W—H2W1	0.85
			
C2—N1—C1	122.55 (14)	C10—N3—H1N3	116.4 (14)
C2—N1—H1N1	118.2 (12)	C6—N3—H1N3	118.4 (14)
C1—N1—H1N1	119.3 (12)	O1—C6—N3	119.75 (16)
C2—N2—H1N2	119.8 (15)	O1—C6—C7	125.67 (15)
C2—N2—H2N2	119.1 (16)	N3—C6—C7	114.58 (15)
H1N2—N2—H2N2	120 (2)	C8—C7—C6	120.84 (16)
C5—C1—N1	119.97 (16)	C8—C7—H7	122.4 (12)
C5—C1—H1	124.8 (12)	C6—C7—H7	116.7 (12)
N1—C1—H1	115.1 (12)	C7—C8—C9	121.09 (17)
N2—C2—N1	118.97 (15)	C7—C8—H8	123.0 (13)
N2—C2—C3	123.30 (16)	C9—C8—H8	115.8 (13)
N1—C2—C3	117.72 (15)	C10—C9—C8	118.78 (15)
C4—C3—C2	120.72 (16)	C10—C9—H9	120.8 (16)
C4—C3—H3	121.2 (13)	C8—C9—H9	120.4 (17)
C2—C3—H3	118.0 (13)	C9—C10—N3	119.51 (15)
C3—C4—C5	118.93 (16)	C9—C10—C11	125.75 (15)
C3—C4—H4	123.4 (11)	N3—C10—C11	114.73 (14)
C5—C4—H4	117.6 (11)	O2—C11—O3	126.89 (15)
C1—C5—C4	120.07 (16)	O2—C11—C10	117.57 (14)
C1—C5—Cl1	119.85 (13)	O3—C11—C10	115.51 (15)
C4—C5—Cl1	120.09 (13)	H1W1—O1W—H2W1	131.4
C10—N3—C6	125.17 (15)		
			
C2—N1—C1—C5	−0.7 (3)	O1—C6—C7—C8	−180.0 (2)
C1—N1—C2—N2	−179.18 (19)	N3—C6—C7—C8	−0.4 (3)
C1—N1—C2—C3	1.8 (3)	C6—C7—C8—C9	0.7 (3)
N2—C2—C3—C4	179.8 (2)	C7—C8—C9—C10	0.3 (3)
N1—C2—C3—C4	−1.2 (3)	C8—C9—C10—N3	−1.5 (3)
C2—C3—C4—C5	−0.5 (3)	C8—C9—C10—C11	176.86 (19)
N1—C1—C5—C4	−1.1 (3)	C6—N3—C10—C9	1.8 (3)
N1—C1—C5—Cl1	178.92 (14)	C6—N3—C10—C11	−176.69 (18)
C3—C4—C5—C1	1.7 (3)	C9—C10—C11—O2	179.9 (2)
C3—C4—C5—Cl1	−178.37 (16)	N3—C10—C11—O2	−1.7 (3)
C10—N3—C6—O1	178.73 (18)	C9—C10—C11—O3	−1.6 (3)
C10—N3—C6—C7	−0.8 (3)	N3—C10—C11—O3	176.80 (17)

### Hydrogen-bond geometry (Å, °)

**Table:**

*D*—H···*A*	*D*—H	H···*A*	*D*···*A*	*D*—H···*A*
O1W—H1W1···O3^i^	0.85	1.85	2.696 (3)	174
O1W—H2W1···O1W^ii^	0.85	1.99	2.732 (5)	145
N1—H1N1···O3^iii^	0.98 (2)	1.67 (2)	2.637 (2)	170 (2)
N2—H1N2···O1^iv^	0.87 (2)	1.97 (2)	2.823 (2)	168 (2)
N2—H2N2···O2^iii^	0.84 (3)	2.04 (3)	2.882 (2)	179 (3)
C4—H4···O1^v^	0.93 (2)	2.39 (2)	3.296 (2)	166 (2)

Symmetry codes: (i) −*x*+3/2, −*y*+1, *z*+1/2; (ii) *x*−1/2, −*y*+1/2, −*z*+2; (iii) −*x*+1/2, −*y*+2, *z*+1/2; (iv) *x*−1/2, −*y*+3/2, −*z*+1; (v) *x*, *y*, *z*+1.
